# The Role of “Bone Immunological Niche” for a New Pathogenetic Paradigm of Osteoporosis

**DOI:** 10.1155/2015/434389

**Published:** 2015-09-30

**Authors:** Danilo Pagliari, Francesco Ciro Tamburrelli, Gianfranco Zirio, Estelle E. Newton, Rossella Cianci

**Affiliations:** ^1^Institute of Internal Medicine, Catholic University of the Sacred Heart, Largo A. Gemelli 8, 00168 Rome, Italy; ^2^Institute of Orthopedics, Catholic University of the Sacred Heart, Largo A. Gemelli 8, 00168 Rome, Italy; ^3^CytoCure LLC, 100 Cummings Center, Suite 430C, Beverly, MA 01915, USA

## Abstract

Osteoporosis is characterized by low bone mass and microarchitectural deterioration of bone tissue. The etiology and pathogenetic mechanisms of osteoporosis have not been clearly elucidated. Osteoporosis is linked to bone resorption by the activation of the osteoclastogenic process. The breakdown of homeostasis among pro- and antiosteoclastogenic cells causes unbalanced bone remodeling. The complex interactions among these cells in the bone microenvironment involve several mediators and proinflammatory pathways. Thus, we may consider the bone microenvironment as a complex system in which local and systemic immunity are regulated and we propose to consider it as an “immunological niche.” The study of the “bone immunological niche” will permit a better understanding of the complex cell trafficking which regulates bone resorption and disease. The goal of a perfect therapy for osteoporosis would be to potentiate good cells and block the bad ones. In this scenario, additional factors may take part in helping or hindering the proosteoblastogenic factors. Several proosteoblastogenic and antiosteoclastogenic agents have already been identified and some have been developed and commercialized as biological therapies for osteoporosis. Targeting the cellular network of the “bone immunological niche” may represent a successful strategy to better understand and treat osteoporosis and its complications.

## 1. Osteoporosis: The Involvement of the Immune System in Osteoclastogenesis and Bone Resorption

Osteoporosis is a systemic skeletal disease, largely characterized by low bone mass and microarchitectural deterioration of bone tissue, leading to increased bone fragility and consequent increase in fracture risk. The etiology and pathogenetic mechanisms of osteoporosis have not been clearly elucidated [1].

Osteoporosis is a major health risk in people over 50. However, to date it is not completely clear whether osteoporosis is a separate bone disease or if it is secondary to the physiological bone aging process.

Osteoporosis is linked to bone tissue loss due to an activation of osteoclastogenic process. Osteoclastogenesis involves several cell subsets, in particular osteoblasts and osteoclasts. The final effector cells that determine bone matrix rearrangement and tissue loss are osteoclasts. Other cell types, such as macrophages and innate adaptive immune cells, also contribute to the tissue microenvironment that orchestrates osteoclastogenic process. In the ideal physiological condition all these cells subsets and their local related mediators are in perfect balance in the bone microenvironment. The result is balanced osteoclast and osteoblast activity and normal bone homeostasis. On the contrary, during osteoporosis and other bone pathological conditions, the harmony between cell subsets and their related mediators in the bone microenvironment breaks down and osteoclast activity increases relative to osteoblast activity. In such a case, activation of bone resorption occurs, resulting in bone tissue loss [2].

Proinflammatory cytokines, especially IL-1, IL-6, IFN-gamma, and TNF-alpha, have been shown to be involved in the pathogenesis of bone resorption in several bone diseases [3]. Proinflammatory T helper cells are now considered potent modulators of bone turnover and are important sources of osteoclastogenic cytokines under inflammatory conditions. It is well known that osteoclasts play a crucial role in osteoporosis [4]. Osteoclasts are specialized bone-resorbing cells regulated by RANKL and macrophage colony-stimulating factor (M-CSF) [5]; these cells are mainly implicated in the development of bone resorption. M-CSF is released by osteoblasts as a result of endocrine stimulation by parathormone and it acts on osteoclasts. M-CSF exerts its function on osteoclasts where it induces differentiation and activates bone resorption with consequent increase of serum calcium levels.

Bone resorption can be caused by three major mechanisms [6]:

1. increasing osteoclast differentiation, activation, and survival;
2. enhancing expression of receptor activator of NF-kappa B ligand (RANKL);
3. inhibiting the bone-forming osteoblast, whilst stimulating osteoclast formation and function.

Functions and differentiation of both osteoblasts and osteoclasts, the principal cells involved in bone metabolism, are regulated by systemic hormones, such as estrogens and parathormone, and Vitamin D, cytokines, and other local tissue factors. Estrogen deficiency is the principal cause of accelerated bone loss in perimenopause and it is linked to the serum levels of particular cytokines, such as IL-1, TNF-alpha, M-CSF, IL-6, and IL-17 [7]. The levels of these cytokine are elevated during perimenopausal estrogen loss and they may potentiate bone resorption through osteoclast recruitment, differentiation, and activation. Several evidences reported the presence of lower calcitonin levels in women compared with men; however, calcitonin deficiency does not seem to be important in age-related osteoporosis [7].

## 2. Bone Destruction: The RANK/RANKL/OPG Pathway and Others

The principal cytokine involved in the osteoclastogenic process is the receptor activator of NF-kB ligand (RANKL). RANKL binding to its receptor RANK expressed on osteoclast precursors is able to activate NF-kB signaling, leading to the transcription of key osteoclastogenic factors [8]. RANKL activity is balanced in normal bone homeostasis by osteoprotegerin (OPG), a decoy receptor for RANKL secreted by osteoblasts and other cells. Experimental introduction of OPG results in a pronounced decrease in bone disruption [9].

The discovery of the receptor activator of nuclear factor-kB (RANK)/RANK ligand (RANKL)/osteoprotegerin (OPG) signaling pathway has permitted a better understanding of bone metabolism and remodeling. This system represents the major regulatory system for osteoclast formation and action, and it is a principal biological regulatory agent of the Tumor Necrosis Factor (TNF) superfamily in bone metabolism [10, 11].

In the late 1990s, the first component identified for a novel pathway regulating bone remodeling was osteoprotegerin (OPG). OPG was shown to encode a novel member of the TNF-receptor family. Overexpression of the OPG gene resulted in high bone mass and marked reduction in osteoclast number and activity [12]. Before that time, in 1980, it was suggested that osteoblasts might be involved in osteoclastogenesis. The nature of this hypothesized “osteoclast activating factor” remained elusive until 1998, when several laboratories independently identified RANKL as a new member of the TNF family of transmembrane and soluble ligands that could bind to OPG. RANK/RANKL/OPG are closely linked with each other in a single biological pathway [13].

RANKL is secreted by activated T cells and represents a crucial link between bone metabolism and the immune system, directly regulating osteoclastogenesis and bone remodeling [14]. Literature data have demonstrated that activated T and B cells can be the cellular source of RANKL for bone resorption in several bone diseases [15]. In most instances, RANKL relies on M-CSF as a cofactor for osteoclast differentiation, but RANKL can stimulate osteoclastogenesis and bone resorption in mice lacking functional M-CSF. Furthermore, no factor or combination of factors have been shown to be capable of restoring bone resorption when RANKL is absent, indicating the dominant role of RANKL in the regulation of bone resorption [16]. Recent experimental data have demonstrated that M-CSF loses its osteoclastogenic potential if it is present alone in osteoclast cultures [17]. On the other hand, when a fragment of bone is added to these cultures, the osteoclastogenic process is activated, thus demonstrating that the presence M-CSF is not sufficient to activate osteoclasts. The addition of the bone to the osteoclast culture provides other proosteogenic factors, such as RANKL, which are required for bone resorption.

Osteoclast precursors, called preosteoclasts, express the surface receptors RANK. Activation of RANK by RANKL promotes the maturation of preosteoclasts into osteoclasts. The activation of RANK in preosteoclasts results in the initiation of several intracellular signal transduction pathways involving NF-kB. After ubiquitination of the signal, NF-kB is released and it can translocate to the nucleus, where it upregulates cofactors that induce osteoclastogenic and proinflammatory transcription factors. Several growth factors, hormones, cytokines, and drugs that influence bone turnover have been shown to influence the expression of RANKL and OPG [16].

On the other hand, OPG protects the skeleton from excessive bone resorption by binding to RANKL and preventing it from binding to its receptor, RANK [18]. OPG expression is regulated by most of the factors that induce RANKL expression by osteoblasts. Although there are contradictory data, in general, upregulation of RANKL is associated with downregulation of OPG, or at least with lower induction of OPG; in this way, the ratio of RANKL to OPG changes in favor of osteoclastogenesis. Many reports have supported the fact that the RANKL/OPG ratio is an important determinant of bone mass and skeletal integrity [19].

Several cytokines can modulate the RANK/RANKL ratio by stimulating the expression of RANKL by immune cells. In particular, TNF-alpha, IL-1, and IL-18 can boost the activating effect of T cells on osteoclasts, because they upregulate RANKL expression on T cells [14].

Moreover, there are controversial data about the role of the well-known proinflammatory cytokine IFN-gamma in modulating bone resorption and homeostasis. In fact, in an* in vitro* model, IFN-gamma was shown to block RANKL signaling and the consequent osteoclastogenesis process; however, in an* in vivo* animal model, the same cytokine was shown to promote osteoclastogenesis process and the consequent bone resorption; thus, IFN-gamma may have, respectively, both destructing and protecting action on the bone [20]. These data lead to the hypothesis that the effect of IFN-gamma on bone homeostasis depends on the local microenvironment in which it is produced.

Another mode of interaction between immune cells and osteoclasts is via the surface receptor CD137, which is capable of antiosteoclastogenic activity. In fact, T cells may communicate with osteoclasts not only through RANK/RANKL interactions but also through CD137/CD137L ones. CD137 is a costimulatory member of the TNF receptor family induced by T-cell receptor activation and it is capable of transducing signals in both directions, through the receptor and into the cell that expresses the ligand. CD137L is expressed on dendritic cells and osteoclast precursors and it has been demonstrated* in vitro *that CD137L ligation suppresses osteoclastogenesis by inhibiting the multinucleation process [21]. Thus, the activation of the CD137/CD137L axis is a way in which T cells may block osteoclastogenesis process.

Considering bone resorption process, we have examined the role of osteoclastogenesis induction that involves osteoclasts activation. However, the same results in bone damage may be occur through the inhibition of osteoblasts activation. In fact, recent* in vivo* experimental data has demonstrated that at the bone level in human osteoporosis fracture there is an increase of osteoblasts inhibitors, such as the Wnt protein family molecules DKK-1 and sclerostin (SOST), and of osteoclastogenesis activators, such as RANKL, M-CSF and TGF-beta; moreover, this study demonstrated that an increase of RANKL/OPG ratio is present, further confirming the association of the RANK/RANKL/OPG axis in the pathogenesis of bone disruption in osteoporosis fractures [22].

## 3. The Breakdown of Cellular Network in the “Bone Immunological Niche”: A Battle among Several Armies

Both Th1 and Th2 cytokine pattern producing cells are linked with bone resorption. In fact, both Th1 and Th2 cells may inhibit osteoclast differentiation by releasing IFN-gamma and IL-4, respectively [2]. The cytokines produced by Th1/Th2 cells mediate osteoclast formation and function and this cellular axis has been shown to be dysregulated in several bone pathologies [2]. Hence, literature data suggest that impairment of T-cell subpopulations and their related cytokine patterns is present in several bone pathologies.

The classic paradigm of Th1/Th2 axis was maintained until 2005, when a distinct lineage of proinflammatory T helper cells, named Th17 cells, was identified [23]. Indeed, it became evident that the T-cell subsets involved in regulation of osteoclasts differentiation are not limited to Th1 and Th2 cells. Th17 cells are characterized by proinflammatory action, the expression of the transcriptional factors STAT-3 and ROR-gammaT, and the production of IL-17. IL-17 is an important proinflammatory factor that is mainly produced by Th17 cells and plays an important role in osteoclast differentiation [24]. Several studies have shown that Th17 cells are increased in many bone diseases and in osteoporosis in particular. Various cytokines, such as IL-6, TGF-beta, IL-23, and IL-1beta contribute to the differentiation and/or amplification of Th17 cells [25–27]. Indeed, it is certain that Th17 cells and IL-17 significantly contribute to the development of bone resorption [2, 28].

In addition, it has been demonstrated that T-cell subpopulations show a functional plasticity determined by the local microenvironment. Thus, microenvironment may modify T-cell function determining a shift from proinflammatory cytokine pattern producing cells to other proinflammatory ones, such as from Th1/Th2 to Th17 cells, or from proinflammatory cytokine producing cells to anti-inflammatory ones, such as from Th1/Th2/Th17 cells to T regulatory cells (Tregs). For example, IL-23 may induce the differentiation of naive T cells into highly pathogenic Th17 cells. Th17 cells produce IL-17 that induces osteoclast function supporting cells, such as synovial fibroblast and osteoblasts, and induces them to express RANKL. IL-17 strongly induces the secretion of TNF-alpha and IL-1 by synovial macrophages and induces osteoclast formation [29]. On the other hand, Th17 cells may directly contribute to bone loss by producing RANKL. In fact, IL-17 stimulates fibroblasts and osteoblasts to produce RANKL [30]. This cytokine is critical for the development of osteoclasts, the major cells responsible for bone erosion. The role of Th17 cells in inducing bone resorption has recently been recognized as mediating systemic bone loss in several inflammatory bowel diseases, such as Crohn's disease [5, 31]. It has been shown that IL-17 not only stimulates RANKL expression in cultures of osteoblasts but also induces osteoclast differentiation. IL-17 directly stimulates human osteoclastogenesis from peripheral blood mononuclear cells (PBMCs) and it also promotes the formation of actin rings in mature osteoclasts [32]. Moreover, Th17 cells play a critical role in the pathogenesis of several bone diseases, such as rheumatoid arthritis, but the mechanisms by which these cells regulate the development of these diseases are not yet fully understood [33].

Additionally, Th17 cells may be a potent osteoclastogenic mediator in estrogen-deficient osteoporosis. In fact, estrogen deficiency promotes osteoclastogenesis by upregulating Th17 cell populations in bone marrow and IL-17 levels in peripheral blood [34]. In postmenopausal women, the production of proinflammatory cytokines is greater than that in premenopausal subjects and it is related to estrogen deficiency [14, 35].

Functions and development of proinflammatory cells are regulated by the activity of several transcriptional factors. STAT-3 is the major transcription factor that is critical for Th17 cell differentiation [36]. STAT-3 protein exists in a latent form in the cytoplasm. STAT-3 becomes phosphorylated on tyrosine residues upon receptor activation by cytokines, such as IL-6, and forms homo- or heterodimers that translocate to the cell nucleus, where they act as transcription activators. Activated STAT-3 translocated into the nucleus promotes the transcription of ROR-gammaT, the essential transcription factor of Th17 cells [37]. STAT-3 also regulates the expression of IL-17, IL-21, and IL-23R, which are all of the utmost importance in the effector function of Th17 cells [28]. STAT-3 is activated in inflamed synovium [38]: in fact, it has been demonstrated that STAT-3 plays essential roles in inflammatory arthritis. STAT-3 is critical in the growth, differentiation, and survival of cells, and it was reported that STAT-3 activation in stromal/osteoblastic cells is required for the induction of RANKL and osteoclast formation [28].

On the other hand, STA-21 is a small molecule with potent STAT-3 inhibiting activity [39]. It impedes STAT-3 DNA binding activity, STAT-3 dimerization, and STAT-3 dependent luciferase activity. It has been demonstrated in a knock-out animal model that STA-21 acts in decreasing the proportion of Th17 cells and of their related proinflammatory cytokines and in increasing the proportion of the anti-inflammatory Tregs [28]. Thus, the activation of STA-21 may reduce tissue inflammation pathways and may further reduce osteoclastic activity and the related bone resorption. Hence, STA-21 could be a promising biological therapeutic agent for several bone diseases.

**Figure 1:**
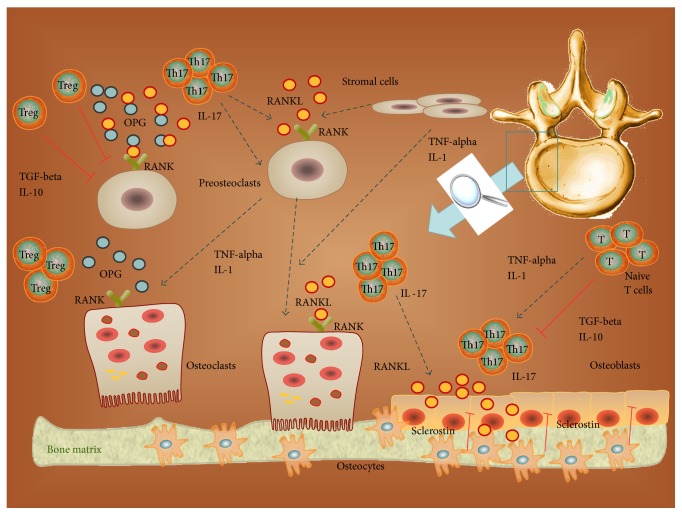
The complex cellular network of the “bone immunological niche.” OPG: osteoprotegerin.

We have extensively examined the potential bone destructing role of several T-cell subpopulations, such as Th1, Th2, and Th17 cells (Figure 1). However, not all T cells promote bone destruction. In fact, T-cell subpopulations can also inhibit osteoclast resorption interacting within the bone microenvironment by the release of cytokines that inhibit osteoclastogenesis, including IFN-gamma, TGF-beta, GM-CSF, IL-4, IL-10, IL-18, and IL-23 [4]. Among these cytokines, as described above in this review, we have to remember that TGF-beta and IFN-gamma may function in a dichotomic way on bone homeostasis. Hence, further* in vivo* studies are needed to better understand the effective role of these cytokines in human bone pathology. Moreover, a new antiosteoclastogenic factor produced by osteoblast lineage has been identified. It is IL-33, a cytokine that influences osteoclast formation. IL-33 is produced by osteoblasts and sporadically by osteocytes. In osteoblasts, it was strongly stimulated by parathormone (PTH) and oncostatin M, two agents that promote bone formation. IL-33 inhibits osteoclast formation* in vitro*, through the induction of other osteoclast inhibitors: GM-CSF, IL-14, IL-13, and IL-10 [40].

Furthermore, literature data have revealed that the newly discovered T regulatory cells (Tregs) [23], a T-cell subpopulation recognized for its anti-inflammatory activity, its role in immune tolerance, the capacity to suppress immune responses, and the expression of the transcription factor FoxP3, take part in bone homeostasis inhibiting osteoclast differentiation by their release of the antiosteoclastogenic cytokines TGF-beta, GM-CSF, IFN-gamma, IL-5, and IL-10 [41, 42]. The potential role of Tregs in modulating bone homeostasis in a FoxP3-overexpressing mouse model characterized by increased numbers of Tregs has been demonstrated; these mice showed high bone volume and low osteoclast numbers, with no change in bone formation showing partial attenuation of bone loss [43].

Nevertheless although there is extensive experimental evidence about the role of Tregs in limiting bone resorption, to date there are no data that describe the impact of these cells in human osteoporosis. So, further studies are needed to better understand the involvement of Tregs in the pathogenesis of disease and their potential role in the developing of osteoporosis biological based therapies.

Some recent studies have demonstrated that cross-talk within the bone microenvironment is not limited to bone cell lineages; in fact, other cells of the immune system may be involved in the regulation of bone homeostasis, such as a particular population of resident tissue macrophages.

Resident tissue macrophages (osteomacs) are a recently identified distinct population of bone resident macrophage cells that function in the bridge between innate and adaptive immune responses. These cells are defined by their expression of the cell surface antigen F4/80 and are usually located close to bone surfaces where they form a canopy over bone-forming osteoblasts. Osteomacs have been found to be associated with bone surfaces but their frequency, distribution, and tissue-specific functional contributions have not been explored. Osteomacs are involved in regulating osteoblast maturation and function and they are important in bone homeostasis and repair [44].

**Figure 2:**
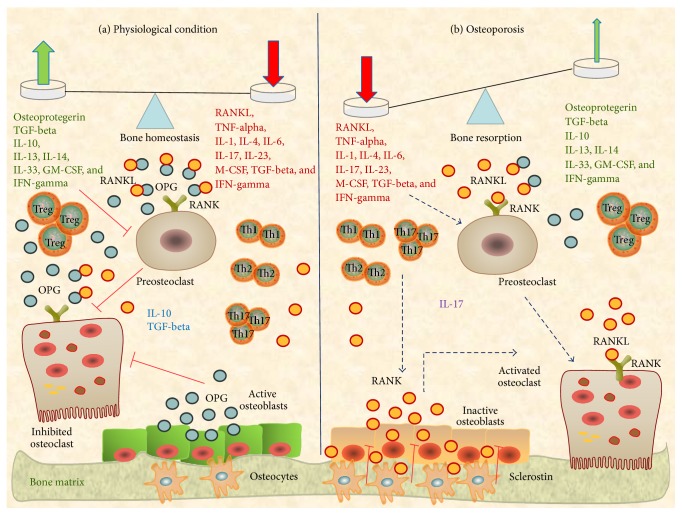
Osteoclastogenic pathways in “bone immunological niche.” At bone tissue level, on one hand, there are the proinflammatory Th1, Th2, and Th17 cells with related proosteoclastogenic cytokines (RANKL, TNF-alpha, IL-1, IL-4, IL-6, IL-17, IL-23, M-CSF, TGF-beta, and IFN-gamma) and other immune cells such as proinflammatory macrophages; on the other hand, there are the anti-inflammatory Tregs with related antiosteoclastogenic cytokines (OPG, TGF-beta, IL-10, IL-13, IL-14, IL-33, GM-CSF, and IFN-gamma) and other immune cells with regulatory functions, such as osteomacs. The bone microenvironment functions as a dynamic model in which a continuous balancing between proosteoclastogenic and antiosteoclastogenic mediators is performed. (a) In physiological condition anti- and proosteoclastogenic factors are in* equilibrium* and bone homeostasis is conserved. (b) In osteoporosis condition, proosteoclastogenic factors prevail and bone resorption and remodeling develop. OPG: osteoprotegerin, GM-CSF: granulocyte-macrophage colony stimulating factor, and M-CSF: macrophage colony stimulating factor.

Normally, in a bone microenvironment characterized by chronic inflammation, macrophages are closely related to bone-resorbing osteoclasts and share a dependence on the lineage-specific growth factor CSF-1. They function as proinflammatory cells producing proinflammatory mediators and cytokines that induce the differentiation of osteoclast from preosteoclasts and the osteoclasts function [45]. This active presence of macrophages in bone tissue raises the question of whether they may be considered a third player in bone homeostasis and turnover. Thus, in such a proinflammatory tissue microenvironment, bone resorption is activated with consequent bone loss. Indeed, the bone microenvironment is characterized for the simultaneous presence of proinflammatory and anti-inflammatory mediators and cells. So, as described above in this review, at bone level, on one hand there are the proinflammatory Th1, Th2, and Th17 cells and other immune cells such as proinflammatory macrophages, and on the other hand there are the anti-inflammatory Tregs and other immune cells with regulatory functions, such as osteomacs. In this manner, the bone microenvironment functions as a dynamic model in which a continuous balancing between proinflammatory and anti-inflammatory mediators is performed. When the side of the scale leans in the proinflammatory sense, bone resorption and remodeling develop; on the contrary, when the side of the scale leans in the anti-inflammatory sense, bone homeostasis is conserved (Figure 2). Thus, we may consider the bone microenvironment as a complex system in which local and systemic immunity is regulated and we propose to consider it as an “immunological niche.”

## 4. Bone Homing Pathways

Rather than hypothesizing a stochastic model in which immunological cells are recruited into bone tissue, we think that bone homing is regulated through several systemic and local mechanisms in the bone microenvironment that involve cytokines, tissue local factors, Toll Like Receptors (TLRs), adhesion molecules and their related receptors, and other innate and adaptive immune cells. Studying the bone “immunological niche” will permit a better understanding of the complex cell trafficking regulating bone resorption and disease. The concept of “immunological niche” has been previously introduced by our group with the aim of explaining the pathogenesis of other inflammatory diseases [27, 46].

Furthermore, as it has been well established for different diseases, a crucial role in the pathogenetic mechanisms is played by Toll Like Receptors (TLRs). TLRs are transmembrane proteins that are typically expressed either on the cell surface or in endosomes. They act as pathogen recognition receptors (PRRs), identifying microbe-associated molecular patterns (MAMPs), that are specific for microbes and essential for their survival. TLR signaling is involved in epithelial cell proliferation, immunoglobulin production, and antimicrobial peptide expression; TLRs are also expressed by other immune cells and can activate an inflammatory response involving both innate and adaptive components [47]. TLRs mediate the interconnection between pathogens and innate immunity [48]. When activated by a pathogen or other specific signals, TLRs determine the initiation of immune response. Dysregulation of TLRs expression is linked to several pathologies, such as bone disease. TLRs are also expressed in osteoclasts and in other cells present at bone tissue, such as macrophages, dendritic cells, and T cells, and their activation affects these cells' differentiation and activity. TLR3, TLR4, and TLR9 have been connected to bone physiology and pathology [49]. Several potential mechanisms have been proposed to explain how bacteria might cause bone destruction. In addition to release of inflammatory cytokines from the immune system, these mechanisms include the release of substances acting directly on the bone matrix, the release of factors capable of directly or indirectly stimulating bone-resorbing cells, and the release of factors capable of inhibiting bone-forming cells, causing apoptosis or other effects. The expression of functional TLRs by osteoclasts raises the possibility of direct modulation of these cells' differentiation and resorptive activity by pathogen-derived TLR ligands [49]. Several studies in animal models have taken in consideration the role of infections in causing bone resorption; hence, it has been shown that bacterial infections are linked to bone loss due to an increase of osteoclastogenesis activity. Nevertheless, these studies do not consider the direct effect of TLRs on osteoclasts activity [50]. Other animal models confirm the role of* bacteria* in bone resorption; in fact, the injection of LPS into mice induces osteoclast differentiation. On the other hand, the activation of TLRs may mediate both activation and inhibition of osteoclastic differentiation and related bone resorption. For example, the activation of TLR9 results in inhibition of RANKL-induced osteoclastogenesis [51], while the activation of TLRs in committed osteoclasts results in increased osteoclastogenesis and is probably the mechanism by which pathogen-induced bone loss occurs. The inhibition of osteoclastogenesis by TLRs activation may play a role in reducing the excessive bone loss caused by pathogenic infection and shifting the balance between the bone and immune systems during infection to recruit immune cells [49].

Moreover, chemokines and other proinflammatory mediators may regulate cellular homing to bone tissue. Multiple cytokines are responsible for increased chemotaxis and homing to the bone marrow.

The interactions between integrins and extracellular matrix (ECM) may modulate bone development and growth as these are strictly regulated by bone microenvironment mediators. The Beta1 subfamily of integrins is the largest integrin subfamily and constitutes the main integrin binding partners of collagen I, the major ECM component of bone [52]. In particular, the interaction of Alpa2/Beta1 integrin with collagen I is a crucial signal for osteoblastic differentiation and mineralization. Emerging evidence suggests that Alpha2/Beta1 integrin can be a major regulator of T-cell activation. In fact, Alpha2/Beta1 integrin may function as receptor for collagen type I on T cells and is expressed only on effector T cells associated with inflammation in extravascular tissues. Hence, Alpha2/Beta1 integrin protects human effector T cells from Fas-mediated apoptosis [53]. In addition, Alpha2/Beta1 integrin is the major collagen-binding integrin expressed by human Th17 cells. It mediates Th17 cell adhesion to collagen, which costimulates the production of IL-17; for this, Alpha2/Beta1 integrin may constitute an important factor for regulating the migration and retention of Th17 cells to the bone [54]. Furthermore, Alpha2/Beta1 integrin can regulate the migration of effector T cells to the tissues that are rich in collagen, such as the bone marrow and the synovium [55]. All these data confirm that Alpha2/Beta1 integrin is a major mediator of cellular bone homing. Finally, integrins are broadly implicated in bone metastasis due to their ability to induce mitogenic intracellular signaling.

In addition, a model of bone homing and cellular trafficking is represented by bone metastasis in human cancer. In fact, bone is a typical metastatic site of several tumors. Literature data reported that integrins mediate mitogenic and migratory signaling of bone homing, whereas the initial attachment of metastatic cells to bone endothelial cells is largely attributed to the lectin class of protein adhesion molecules. It has been demonstrated that cancer cells may modify their surface molecules in order to permit them to achieve bone metastatic tissue targets. Several molecular mechanisms have been elucidated endowing bone metastatic circulating tumor cells with the ability to attach and invade bone tissue. Broad classes of stromal interactions, such as integrin- and lectin-mediated attachment or protease-dependent invasion have been characterized [9]. It has been established that bone metastatic cells can express chemokine receptor 4 (CXCR4) that provokes actin polymerization and pseudopodia formation resulting in migration upon exposure to bone endothelial cell secretions; thus, CXCR-4 enables circulating cancer cells to migrate into bone tissue to form the pre-metastatic niche [56].

Three major integrins are linked to bone seeding of metastatic cells: integrins AlphaV/Beta3, Alpha2/Beta1, and Alpha4/Beta1. AlphaV/Beta3 integrin functions in bone metastasis through binding either osteopontin (OPN), bone sialoprotein (BSP), or CD44. OPN is a major extracellular component of multiple bone tissue cells, and engagement by AlphaV/Beta3 integrin results in MEK induced upregulation of matrix metalloproteinase 9 (MMP9). BSP is a distinct AlphaV/Beta3 ligand expressed in normal bone tissue that is upregulated in bone metastatic lesions compared to metastases in other organs [57]. Then, Alpha2/Beta1 and Alpha4/Beta1 integrins bind collagen I (COL1) and VCAM1 in bone metastasis. COL1 is the major structural component of the bone matrix whereas VCAM1 is constitutively expressed by bone endothelial cells; when bound by Alpha2/Beta1, subsequent RhoC activation primes cell morphology for invasion [58, 59]. Multiple* in vitro* studies have demonstrated that Beta1 integrins are crucial regulators of osteogenesis and mineralization, whereas* in vivo* studies have revealed only mild and sometimes contradictory results on the use of Beta1 integrins in bone repair [52].

Moreover, the initial attachment of metastatic cells to bone endothelial cells is largely attributed to the lectin class of protein adhesion molecules. Glycosylated ligands present on circulating tumor cells, such as PSGL1 and CD44, engage endothelial selectin (SELE) and mediate initial cell attachment and subsequent rolling along bone endothelial cells [60].

The study of cellular trafficking and bone homing may help to identify additional potential pathways to develop new biological therapeutical strategies for osteoporosis. For example, in the field of regenerative medicine, the knowledge about bone homing has been utilized to develop drugs directly able to arrive on their targets in the bone. Hence, regenerative medicine strategies include the use of vehicles for drug delivery to the bone in order to permit the specific action of these drugs on target tissue sites. For example, hydrogels, such as polyethylene glycol (PEG), are water-swollen cross-linked polymer networks that offer significant advantages as vehicles for protein delivery due to their high cytocompatibility, low inflammatory profile, biofunctionality, and injectable delivery method [61]. PEG hydrogels are widely used in FDA approved therapeutic products as covalent modifiers of proteins and lipids [62].

Moreover, a potential use of biological vehicles may be driving bone morphogenetic protein (BMP) therapies to bone targets. BMP therapy has emerged as a promising alternative to autografts and allografts. In fact, BMP therapy has been shown to be successful in stimulating bone repair. It has recently been demonstrated in a mouse model that an Alpha2/Beta1 integrin-specific PEG hydrogel BMP-2 carrier may allow better clinical results in inducing bone repair than traditional therapies [61].

## 5. Traditional Therapies and New Osteoporosis Therapeutical Approach Based on Biological Agents

At present, the main aim of osteoporosis treatment is to preserve bone mass and prevent fractures. Despite this, exploration into the disease mechanisms might lead to the development of discoveries of new or improved therapies.

As explained in this review, there is a close cross-communication among osteoblasts, osteoclasts, macrophages, and innate and adaptive immune cells; this cellular network is essential for bone homeostasis. Hence, studying this cellular cross-communication and its interference with the “bone immunological niche” may help to introduce new biologically based therapies for bone diseases.

To date, the more common therapies for osteoporosis are those that act to reduce bone resorption. This goal can be achieved both by osteoclast inhibition therapy and by osteoblast stimulation therapy. Among osteoclast inhibition therapies, two drugs have shown efficacy; these are bisphosphonates and the monoclonal antibody Denosumab (anti-RANKL antibody). Additional pharmacological agents have demonstrated promise in preclinical experiments but have to be tested in clinical trials [9].

Bisphosphonates act in diminishing bone resorption; they became a standard of care in the treatment of osteoporosis. Bisphosphonates, such as zoledronic acid, pamidronate, or ibandronate, specifically bind to the bone matrix and are internalized by osteoclasts upon resorption. Once internalized, these drugs inhibit various metabolic processes such as prenylation and lead to apoptosis [63].

Denosumab is a fully human monoclonal antibody inhibiting RANKL; it is recommended for the treatment of osteoporosis, bone metastases, multiple myeloma, and giant cell tumor of bone. Denosumab inhibits the maturation of preosteoclast into osteoclasts by binding and inhibiting RANKL. This mimics the natural action of the endogenous RANKL inhibitor osteoprotegerin that results decreased in osteoporotic patients. This protects bone from degradation and helps to stabilize the progression of the disease [13, 64].

Compared to bisphosphonates, Denosumab and recombinant OPG demonstrate higher clinical efficacy by targeting RANKL (Table 1). Both have shown similar promise in clinical trials [9].

In this review we have already examined the role of Th17 cells in inducing osteolysis and bone resorption. The molecule STA-21 is able to block the principal Th17 cell transcription factor STAT-3 that activates the transcription of proinflammatory cytokines genes. Thus, the activation of STA-21 may reduce tissue inflammatory patterns, osteoclastic activity, and the consequent bone resorption. Hence, STA-21 could be a promising biological therapeutical agent for several bone diseases. In fact, recent experimental data performed in an* in vivo* mouse model and in an* ex vivo* human model have demonstrated the potential therapeutic role of STA-21; treatment with STA-21 induced the increase of bone protective T regulatory cells (Tregs) and reduced the number of bone destructive Th17 cells and their related production of proinflammatory IL-17 [28]. Considering that Th17 cell maturation, growth, and proliferation are dependent on a various number of cytokines as described in this review will help to introduce new biological therapies for bone remodeling which utilize these cytokine patterns.

It has been shown that Alpha2/Beta1 integrin is the major collagen-binding integrin expressed by human synovial proinflammatory and proosteoclastogenic Th17 cells and it is the pivotal mediator of lymphocytes bone homing. An* in vivo* mouse model has recently demonstrated that blocking Alpha2/Beta1 integrin with a specific monoclonal antibody led to a decrease in the number of Th17 cells in the joint and to a reduction of IL-17 levels in mice; this was associated with a reduction of bone loss due to an inhibition of RANKL levels and osteoclast numbers and activity [3]. Thus, Alpha2/Beta1 integrin may be a promising mediator to develop new drugs for biological therapies of human osteoporosis.

In conclusion, studying the complex cellular network engaging bone and immune cells responsible of the pathogenesis of osteoporosis has permitted the individuation of new pathological mediators, mechanisms and pathways. This cellular interplay involves both circulating and tissue cells, and it is strictly regulated by the complex microworld of the “bone immunological niche.” However, further data are needed to better explain some other pathogenetic disease mechanisms. There are several potential warriors in this disease battle, such as osteoblasts, osteoclasts, macrophages, and innate and adaptive immune cells. The breakdown of the homeostasis among these warriors causes unbalanced bone remodeling. The goal of a perfect therapy may potentiate good warriors and block bad ones. In this scenario, additional factors may take part in helping or hindering the warriors. Hence, several proosteoblastogenic and antiosteoclastogenic agents have been individuated and some of them have been developed and commercialized as biological therapies for osteoporosis. Many others are currently under investigation. Thus, targeting the cellular network of the “bone immunological niche” may represent a successful strategy to better understand and treat osteoporosis and its complications.

## Figures and Tables

**Table 1:** Traditional and potential therapies for osteoporosis.

Molecular target	Mechanism of action	Approved agent
Prenylation	Osteolysis inhibitor	*Zoledronic acid and other bisphosphonates*
RANK/RANKL/OPG axis	Osteoclastogenesis inhibitor	*Denosumab*
Recombinant osteoprotegerin (OPG)	Osteoclastogenesis inhibitor	
STA-21 (STAT-3 inhibitor)	Osteoclastogenesis inhibitor	
TGF-beta	Osteoclastogenesis inhibitor	
Alpha2/Beta1 integrin	Bone homing and drug vehicles	
